# Cohen syndrome and early-onset epileptic encephalopathy in male triplets: two disease-causing mutations in *VPS13B* and *NAPB*

**DOI:** 10.1007/s10048-023-00710-2

**Published:** 2023-02-13

**Authors:** Alice AbdelAleem, Naim Haddad, Ghada Al-Ettribi, Amy Crunk, Ahmed Elsotouhy

**Affiliations:** 1 Neurogenetics Research Lab, Weill Cornell Medicine Qatar, Doha, Qatar; 2 Clinical Genetics Division (Clinical Privilege), Hamad Medical Corporation, Doha, Qatar; 3 Present Address: Medical Molecular Genetics Department, Human Genetics and Genome Research Institute, National Research Centre, Cairo, Egypt; 4 Neurology Department, Weill Cornell Medicine Qatar, Doha, Qatar; 5 GeneDx, Inc, Gaithersburg, MD USA; 6 Neuroradiology Department, Hamad Medical Corporation, Doha, Qatar

**Keywords:** Autism, Cohen syndrome, Multifocal epilepsy, *NAPB*, Sexual arousal, SNARE complex, *VPS13B*

## Abstract

Cohen syndrome (CS) is a rare multisystem autosomal recessive disorder associated with mutations in *VPS13B* (vacuolar protein sorting homolog 13B). The *NAPB*-related neurodevelopmental disorder is characterized mainly by early-onset epileptic encephalopathy (EOEE) and is associated with mutations in *NAPB* that encodes for SNAP-beta (soluble NSF attachment protein beta). Here we describe male triplets, clinically presenting with the phenotype of subtle but distinctive facial features, intellectual disability, increased body weight, neonatal EOEE, and prominently variable abnormal behaviors of autism and sexual arousal. The EEG showed multifocal epilepsy, while the brain MRI showed no abnormalities. Diagnostic exome sequencing (ES), the applied next-generation sequencing approach, revealed the interesting finding of two novel homozygous variants in two genes: *VPS13B* missense variant (c.8516G > A) and *NAPB* splice-site loss (c.354 + 2 T > G). Sanger sequencing verified the segregation of the two recessive gene variants with the phenotype in family members. The prediction algorithms support the pathogenicity of these variants. Homozygosity mapping of ES data of this consanguineous family revealed multiple chromosomal regions of homozygosity stretches with the residing of *VPS13B* (chr8: 100830758G > A) and *NAPB* (Chr20: 23,375,774 A > C) variants within the largest homozygous blocks further supporting the disease-genes causal role. Interestingly, the functions of the two proteins; VPS13B, a transmembrane protein involved in intracellular protein transport, and SNAP-beta involved in neurotransmitters release at the neuronal synaptic complexes, have been associated with Golgi-mediated vesicular trafficking. Our ES findings provide new insights into the pathologic mechanism underlying the expansion of the neurodevelopmental spectrum in CS and further highlight the importance of Golgi and Golgi-membrane-related proteins in the development of neurodevelopmental syndromes associated with early-onset non-channelopathy epilepsy. To our knowledge, this is the first report documenting multifocal EOEE in CS patients with the association of a pathogenic *NAPB* variant.

## Introduction

Cohen syndrome (CS) (OMIM #216,550) is a rare autosomal recessive multisystem disorder, characterized by a spectrum of global developmental delay, intellectual disability, hypotonia, facial dysmorphism, ocular defects, neutropenia, endocrine and skeletal abnormalities [1, 2]. Associated seizures and behavioral disturbances were described in a few cases of CS, in which cortical brain malformation or hippocampus atrophy was demonstrated [3, 4]. Clinical heterogeneity in CS was evident among patients from different ethnic backgrounds [5], genetic isolates as well as patients of the same ancestry [6]. The syndrome has been described in diverse ethnicities, but in very few Arab families: Omani, Lebanese, and Tunisian [5, 7]. Investigators relate the heterogeneity in the appearance of certain clinical features involving short stature, delayed puberty, blindness on top of retinal dystrophy, or neutropenia to the ethnic background of the patients’ population [8]. Mutations in the vacuole protein sorting 13B (*VPS13B*) gene, also known as *COH1* [OMIM *607817], are recognized to underlie the characteristic features in CS [5]. VPS13B protein, one of the four members of the human VPS13 protein family that share a common feature of being giant proteins > 300 KD in size, is a Golgi peripheral membrane protein that co-localizes with Golgi matrix proteins. Silencing of *VPS13B* in vitro and in CS patients’ fibroblasts revealed fragmentation and disorganization of Golgi structures, hence its suggested roles in maintaining Golgi integrity and intracellular trafficking of Golgi-derived vesicles [9].

On the other hand, mutations in the *NAPB* gene [N-ethylmaleimide-sensitive factor (NSF)-attachment protein beta, OMIM *611270] that encodes for the SNAP-beta, a protein component of the SNARE (SNAP-receptor) complex, have been described in association with early-onset epileptic encephalopathy (EOEE) characterizing the multifocal intractable seizures in disorders of neurodevelopmental nature [10–13]. SNAP-beta plays a basic role in the fusion/priming of neuronal synaptic vesicles and the release of neurotransmitters [10].

This study aims to find out the genetic defect underlying the unexplained phenotype of intellectual disability, mild dysmorphism, developmental delay, EOEE, and prominent behavioral patterns in male triplets. Herein, we report the interesting genetic findings identified in Palestinian male triplets, who were found to carry novel homozygous recessive variants in *VPS13B* and *NAPB*, and present with CS phenotype, EOEE, and variably prominent behavioral abnormalities.

## Subjects and methods

### Subjects

Six subjects, three male probands (single-placenta triplet) and three family members (mother, father, and a healthy sibling), were subjected to diagnostic exome sequencing genetic testing and analysis. The male triplets were on follow-up in the Neurology Clinic, Hamad Medical Corporation, Qatar, and first attended the Genetic Clinic, at the age of 23 years. Written informed consent was obtained from the participants and patients’ guardians for the data release, capturing photos, and publication.

### Methods

In-house clinical investigations, complete blood pictures, EEG, brain imaging (MRI), and fragile-X testing (outsourced) were performed. Blood samples of the six family members were collected and sent to GeneDx for diagnostic exome sequencing (ES).

#### Exome and Sanger sequencing

Diagnostic exome sequencing (ES) was carried out at GeneDx, using genomic DNA from the probands, parent, and a healthy sibling. The exonic regions and flanking splice junctions of the genome were captured using the IDT xGen Exome Panel v1.0. Massively parallel (NextGen) sequencing was done on an Illumina system with 100-bp paired-end reads. Reads were aligned to human genome build GRCh37/UCSC hg19 and analyzed for sequence variants using a custom-developed analysis tool. Sanger sequencing was applied to verify the segregation of *VPS13B* and *NAPB* variants with the phenotype in all participants of the family members.

#### Prediction algorithms

VarSome,^1^ the human genomic variant search engine [14], the Ensembl variant Effect Predictor^2^ web tool, and GeneDx variants assertion criteria were applied for variants’ interpretation. Variants classified as pathogenic or variant of uncertain significance (VUS) according to the American College of Medical Genetics and Genomics (ACMG) guidelines [15].

#### Homozygosity mapping (HZM)

HZM is a powerful tool for the prioritization and identification of recessively inherited disease-causing genes; the tool provides further supporting evidence for the candidature of the two gene variants. BAM-sequencing files of the proband A, parent, and healthy sibling were combined in one VCF, uploaded, and run on the HomozygosityMapper online tool. Gene Distiller functionality help to allocate the genes in the query (*VPS13B* on chromosome 8 and *NAPB* on chromosome 20) along the homozygous regions.^3^ HZM aims to promote the genetic diagnosis’s reliability by looking at the homozygous stretches on the two chromosomes of interest, Chr8 and Chr20, and whether the corresponding gene was allocated within such homozygous blocks.

## Results

### Clinical presentation

**Fig. 1:**
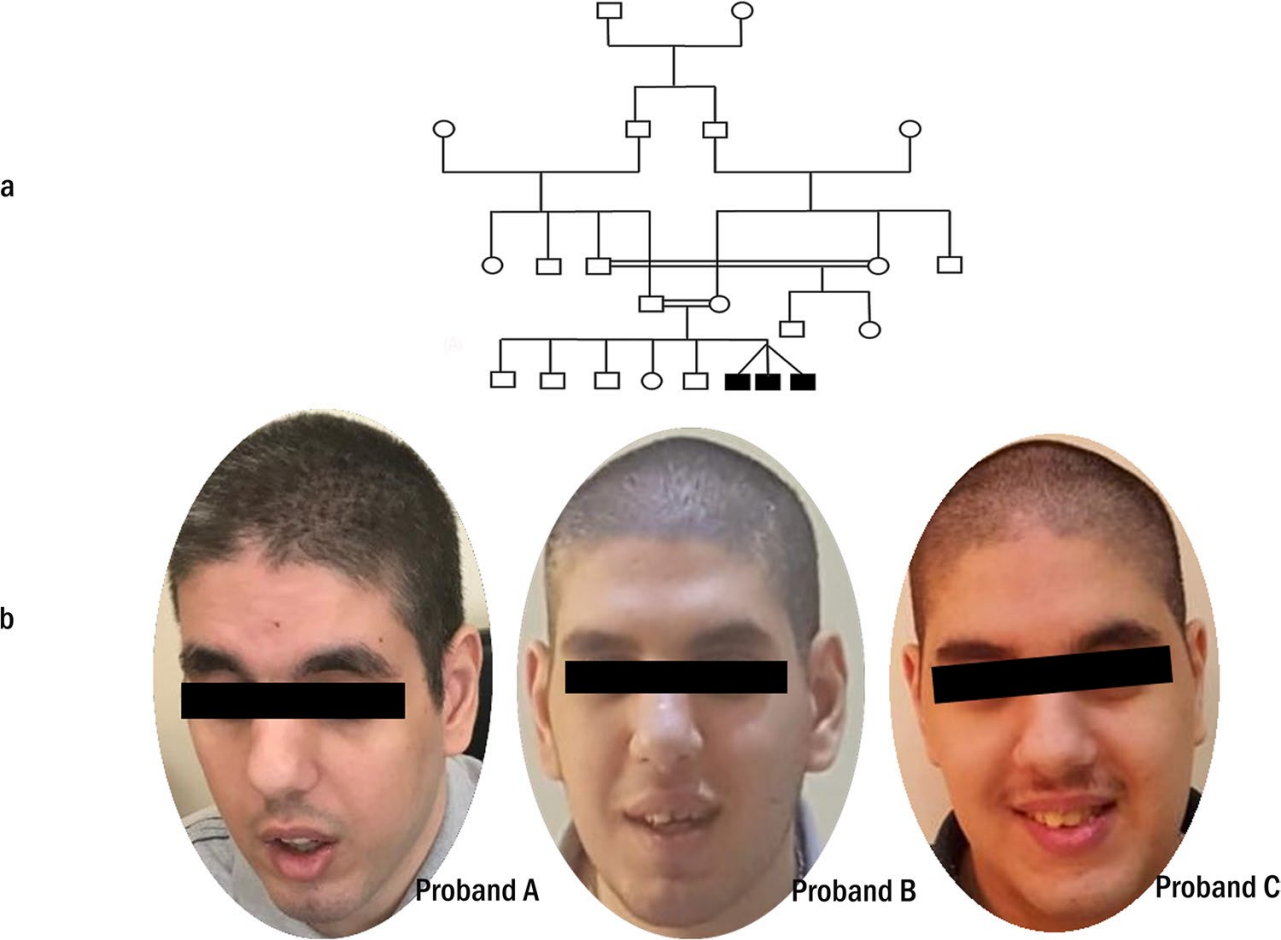
Family pedigree and patients’ photos. **a** First cousin consanguineous Palestinian family, filled rectangular denotes affected male triplets, empty circles and boxes denote healthy subjects. **b** Facial appearance of the male triplets at the age of 23 years shows long face, heavy eyebrows, prominent nose and bulbous nasal tip, short philtrum, open mouth with thick lower lip, long ears, and short neck

A triplet of male probands was born preterm at 36 weeks gestational age by caesarian section. The triplets had a single placenta and were blood group O + . The first-cousin parent has five other older normal offspring (Fig. 1a). The neonatal history described generalized hypotonia, limited spontaneous movements, but no feeding difficulties. Facial characteristics involved notably large, long, and protruding ears, bulbous nasal tip, and triangular face. Motor developmental delay was marked; head support was achieved at the age of 10 months, sat unsupported at 15 months, and walked at two and half years old. Psychomotor retardation and learning disabilities were evident but variable across the three probands (Table 1). At the first genetic assessment (age of 23 years), the probands displayed a facial gestalt of a long face, brushy and heavy eyebrows, long eyelashes, unilateral ptosis of the left eyelid, open mouth with the thick lower lip, short philtrum, short neck, long but not-protruding ears, bulbous nasal tip, and prominent nose (Fig. 1b). All probands showed joint hyperextensibility, bilateral big toes, wide space between the first and second toes, and cylindrical fingers. A history of being overweight as an adolescent was reported. The features of the male gender, long face, cognitive impairment, and psychomotor delay suggested fragile X syndrome as the first provisional clinical diagnosis; however, FMR1-CGG repeats testing proved normal.

**Table 1:** Summary of the clinical characteristics in Palestinian male triplets with Cohen syndrome and EOEE-related neurodevelopmental disorder

Clinical features	Proband A	Proband B	Proband C
Neonatal Birth Weight/Length/and Head circumference	2.300 kg/44 cm/33 cm	2.250 kg/44 cm/32 cm	2.200 kg/45 cm/31 cm
Growth charts at 23 years old:			
Head circumference	56 cm (50th centile)	56 cm (50th centile)	55 cm (below 50th centile)
Stature	171 cm (below 75th centile)	171 cm (below 75th centile)	171 cm (below 75th centile)
Weight*	74 kg (below75th centile)	86 kg (below 90th centile)	66 kg (between 50 and 25th centile)
Neonatal history:			
Hypotonia	+ generalized	+ generalized	+ generalized
Spontaneous movements	Limited	Limited	Limited
Feeding difficulties	−	−	−
Feeding: breast/bottle/or combined	Combined	Combined	Combined
Developmental delay:	Remarkable	Remarkable	Remarkable
Head support	10 months	10 months	10 months
Sit unsupported	15 months	15 months	15 months
Walking	2½ years	2½ years	2½ years
Speaks	3 years	3 years	Never
Puberty	14 years	14 years	14 years
Motor movements and gait	Clumsy/unsteady	Clumsy/unsteady	Clumsy/unsteady
Learning disabilities	Moderate	Moderate	Severe
Verbal communication	+ (speech content not well developed)	+ (speech content not well developed)	−
Behavioral disturbances:	+	+	+ most prominent
Autism or autistic spectrum	−	Autistic spectrum	Autism (full picture)
Sexual arousal	−	−	+ +
Maladaptive	+	+	+
Aggressiveness	−	+	+
Friendly social attitude	+	+	+
Epilepsy:	+	+	+
Onset	6 months	6 months	7 months
During sleep/wakefulness/or both	Sleep (almost always)	Both	Wakefulness
Severity/frequency	Severe/frequent and in cluster	Moderate/less frequent	Mild and occasional
Antiepileptic medications	Lamotrigine, levetiracetam, and escalating dose of clobazam	Clonazepam, lamotrigine, and valproic acid	“Carbamazepine and levetiracetam”
Controlled/uncontrolled	Uncontrolled	Controlled	Very well controlled
EEG	Multifocal	Multifocal	Multifocal
Neutropenia	−	−	−
Imaging:			
Brain MRI	Normal brain images	No available image	No available image
Chest X-ray	Bilateral cervical ribs	Unilateral cervical rib	No available image
Cardiac Echo	Normal	Normal	Normal
Fundus examination^#^	Normal, quite posterior segment	Normal, quite posterior segment	Normal, quite posterior segment
Other features:			
Big first toes, wide space between 1st and 2nd toes	+	+	+
Grayness and whiteness of scalp hair	+	+	−
Generalized joint hyperextensibility	+	+	+
Cylindrical fingers	+	+	+
Hypothyroidism	−	−	+ (at age of 11 years)

+ present, − absent. *The triplet were originally described to be overweight (on 95–98th centiles); swimming and physical activities help weight reduction. ^#^Visual acuity clinically seems fine, but was not possible to assess

#### Epilepsy and behavioral disturbances

**Fig. 2:**
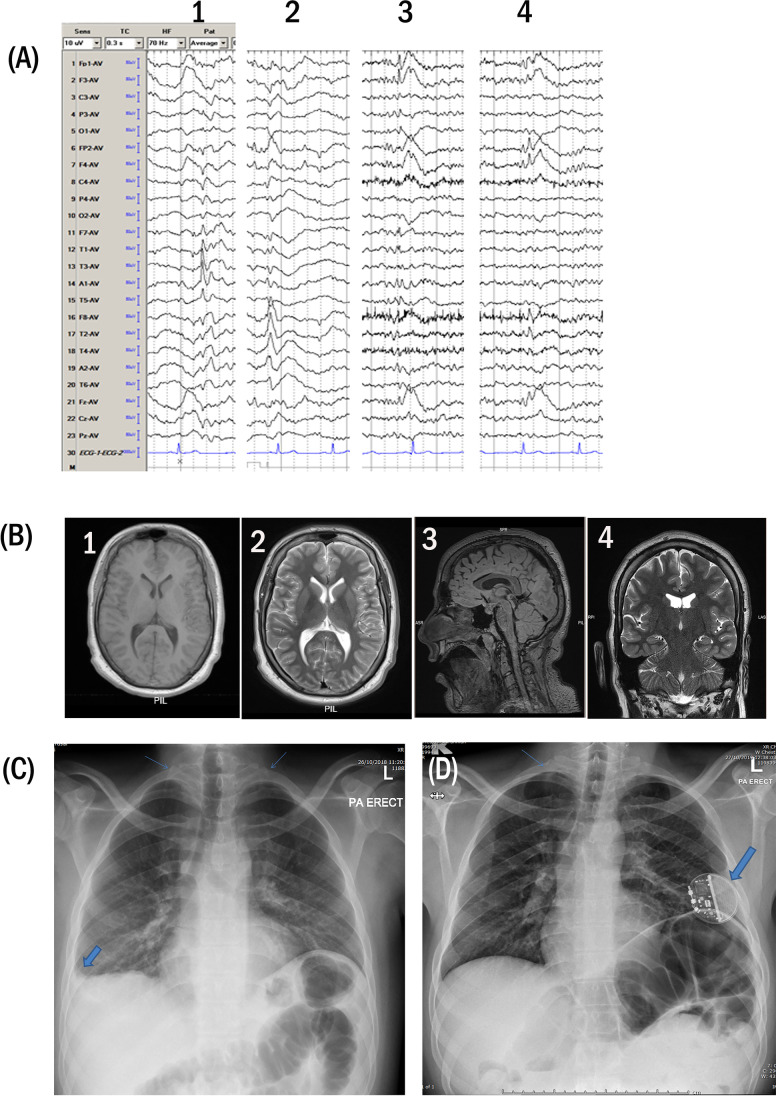
Brain MRI, EEG-recording, and chest X-ray. **a**) Proband A, interictal EEG samples in a referential montage showing multifocal independent sharp waves and spikes: 1, left temporal. 2, right temporal. 3, left frontal. 4, right frontal. **b**) Proband B, axial T1 (1) and T2 (2) brain MR images at the level of the basal ganglia showing no significant abnormality. Sagittal FLAIR (3) and coronal T2 (4) images showing normal size of the corpus callosum and comparable temporal lobes especially the hippocampal regions with no signal abnormality. **c**) and** d**) Plain PA-radiography of the chest; **c** proband A, blunting of the right costophrenic angle (thick arrow) with clear left one. Bilateral bony cervical ribs are seen longer on the right side (blue thin arrows), **d** proband B, small right sided bony cervical rib (thin arrow), left sided subcutaneous VNS battery noted

Epilepsy was a prominent neonatal feature that variably progressed in the triplet. In proband A, the seizures occurred usually during sleep; the patient would awaken looking scared or in discomfort, may scream with loss of awareness, stiffen diffusely, and may exhibit a head turn and bilateral jerking movements. His seizures were frequent, occurring daily, sometimes in clusters with the attacks typically lasting for a minute. The patient is currently on lamotrigine, levetiracetam, and an escalating dose of clobazam with only partial improvement. He failed many other trials of anti-seizure drugs, and specifically developed symptomatic hyperammonemia on valproate. The VEEG (video electroencephalogram) data with interictal and ictal recordings support multifocal epilepsy (Fig. 2A). Despite the protracted nature of the seizures, his brain magnetic resonance imaging was normal (Fig. 2B). Proband B seizures were less frequent, occurring during wakefulness and sleep, and fit the description for classical generalized tonic–clonic (GTC) seizures that last for a few seconds. A more subtle seizure type was sometimes noted with a sudden slowing in speech fluency, echolalia, and blank stare for a minute or less. At the age of 16 years, a vagal nerve stimulator device (VNS) was implanted. At 23 years of age, the VNS was turned off because of suspected worsening sleep apnea. His seizures came under good control on valproic acid after it was added to lamotrigine and clonazepam. A childhood EEG recorded the presence of multifocal epileptiform discharges. Proband C had the mildest presentation of epilepsy; his seizures were well controlled by a combination of antiepileptic medication (Table 1) and became quite infrequent.

#### Behavioral abnormalities

Behavioral abnormalities were particularly significant in proband B, who has the full clinical picture of autism, as well as exaggerated sexual arousal, and maladaptive behaviors. Proband C showed a much milder spectrum of autism, while proband A displayed no recognizable features of autism.

#### Skeletal abnormalities

The cervical ribs, seen in the available radiographs of probands A and B, is a newly reported radiological finding in CS (Fig. 2C and D). Spinal deformities or clubfoot was not identified in any of the triplets.

### Molecular genetic findings

**Fig. 3:**
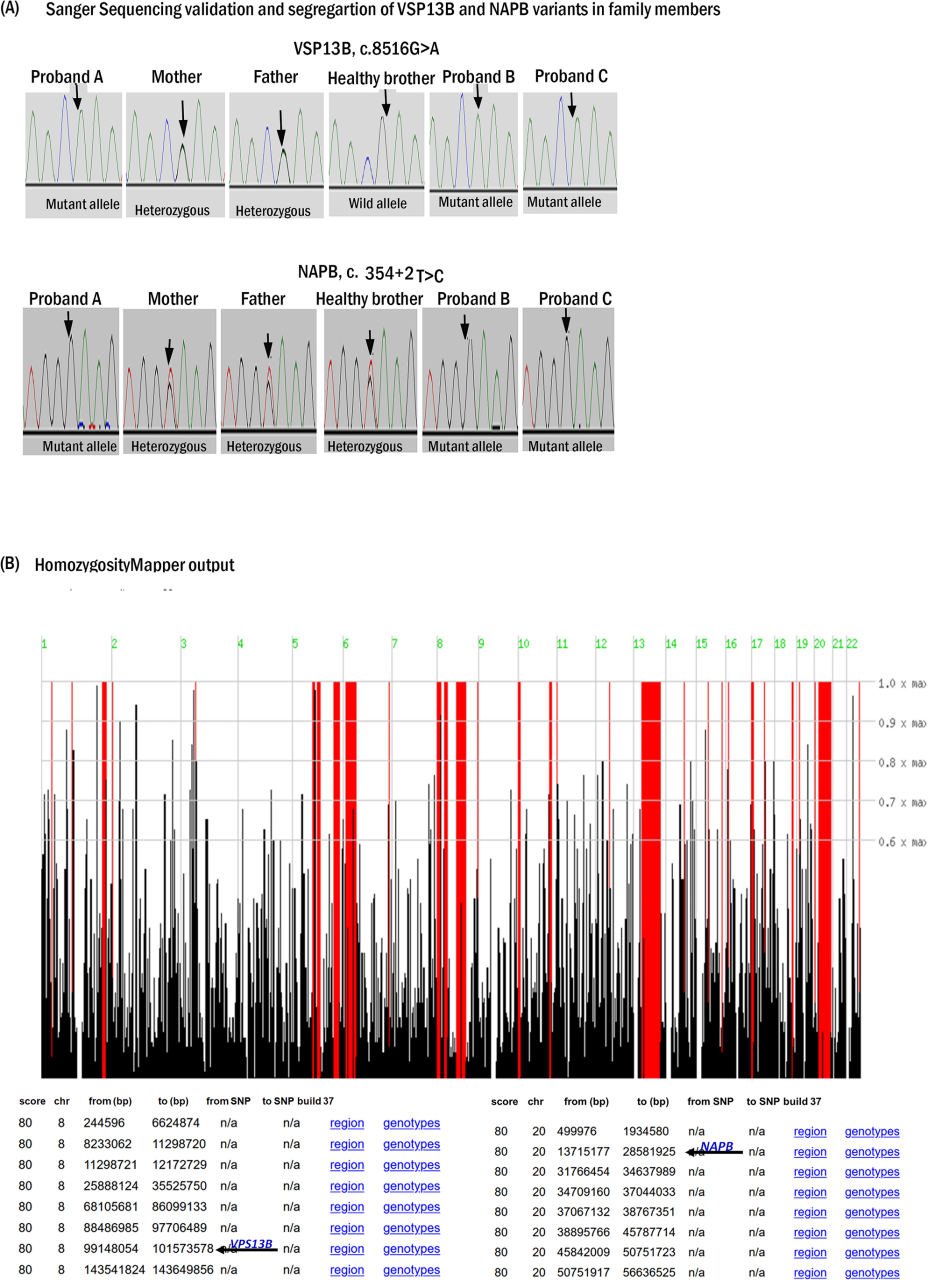
Sanger sequencing and HZM. **a**) Sanger validation and segregation of VPS13B and NAPB variants. Sanger sequencing showing the recessive co-segregation of the two variants in *VPS13B* and *NAPB* genes with the phenotype. **b**) Homozygosity mapping output of the combined ES-VCF. Upper panel: Demonstrating the extensive homozygous stretches on most of the chromosome. Lower panel: The genomic position of the homozygous stretches on chromosomes 8 and 20, the arrows point to the *NAPB* (Chr20: 23,375,774 A > C) and *VPS13B* (100830758G > A) gene variants residing within the homozygous stretches on chromosomes 20 and 8, respectively

ES results validated by Sanger sequencing revealed two homozygous novel variants: a missense variant of uncertain significance, c.8516G > A, p.(Arg2839Gln) in exon 46 (NM_017890.4) of *VPS13B* and a splice-site loss pathogenic variant c.354 + 2 T > G in exon 4 (NM_001283018.1) of *NAPB*. The triplets were homozygous for both variants in *VPS13B* and *NAPB*, while the parent was heterozygous for these variants. The healthy sibling was a carrier for the *NAPB* variant but did not carry the *VPS13B* variant (Fig. 3A).

#### In silicopredictive tools’ findings

The *VPS13B* missense variant is a strong Cohen-causing candidate: variant pathogenicity showed as damaging/pathogenic/or disease-causing in 12 prediction algorithms and as tolerated in only seven. The ACMG classified this variant as VUS based on the pathogenicity score, multiple computational tools that support a deleterious effect on the gene, and the very rare allele frequency in gnomAD exomes (= 0.0000159). The present missense *VPS13B* variant affects the same codon (c.8516) that has been previously altered by a nonsense variant (p.R2839X) in two independently reported CS patients [16]. The arginine, a polar-basic residue at the protein position P.2839, is highly conserved among species (phyloP100way = 9.6) and is replaced here by glutamine, a polar but neutral amino acid. The variant supporting criteria involve being observed in a homozygous status in the affected triplets, the phenotypic fit (the gene is highly specific for the disease presentation), and a high CADD score of 33. Our variant was reported on the CliniVar submission page by another independent clinical testing submitter.

*NAPB* (NM_001283018.1): c.354 + 2 T > G, a splice site loss variant, is a strong candidate for the NAPB-related neurodevelopmental disorder. Regarding variant pathogenicity, it has been shown as damaging/pathogenic/or disease-causing in seven prediction tools and has not been reported as tolerated in any computational prediction tools. The clinical significance of this variant was interpreted as “pathogenic” based on the criteria of being a “null” variant (very strong), not found in any available population databases, and has a strongly conserved position (phyloP100way = 9.09). This variant has the supportive criteria of being homozygous in the triplets and the gene is highly linked to EOEE neurodevelopmental disorder. The *NAPB* variant decreases the splicing efficiency by 80% as predicted by MaxEntScan; there is no detectable nearby alternative splice site that could result in exon skipping. This variant has a CADD score of 34.

The two gene variants co-segregate in a recessive pattern with the family phenotype (Fig. 3A).

#### Homozygosity mapping

ES-BAM files, uploaded on the HomozygosityMapper online tool revealed extensive homozygous stretches on most of the chromosomes. *VPS13B* variant (chr8: 100830758G > A) and *NAPB* variant (Chr20: 23,375,774 A > C) were residing within one of the large homozygous blocks identified on chromosomes 8 and 20, respectively (Fig. 3B).

## Discussion

This report is the first to describe Cohen syndrome (CS) in probands of Arab-Palestinian origin. Clinical heterogeneity in CS presentation is well-acknowledged [5, 6]; however, CS has been reported in very few families of Arab ethnicity [7, 17]. Global developmental delay, learning disabilities, normal brain imaging, and distinctive facial gestalt were featured in the present triplets, in whom the CS diagnosis was first uncovered when they were 23 years old following a diagnostic ES. Features of neonatal/infant feeding difficulties, postnatal microcephaly, short stature, delayed puberty, myopia, retinal dystrophy, and neutropenia were absent in our patients. Earlier studies described neutropenia as an exclusive Caucasian feature and suggested a normal neutrophil count in CS patients of non-European descent [16]. The absence of neutropenia seemed to be a feature in Arab CS patients [16, 7, this report]. In terms of intellectual abilities, our triplets demonstrated variable degrees of intellectual impairment; while proband C had a profound intellectual disability, probands A and B exhibited moderate learning disabilities. This supports previous studies involving patients from different descends [18, 19].

The unique clinical observations in our patients involved the remarkable variability in the abnormal behavioral patterns and the early-onset (neonatal) protracted epilepsy. Autistic behavior in CS was acknowledged in some studies [6, 18, 20], while described as a rare event or non-observed in others [5]. Variability in the autistic association was demonstrated among unrelated patients of different series; however, in the present study, the variability was observed among three triplets that had a single placenta and were of the same gender and blood group suggesting the likelihood of being monozygotic. Proband C showed the full picture of autism in addition to significant sexual arousal and aggressive behaviors. Proband B had a mild spectrum of autism, while proband A showed no clinical features suggestive of autism. The partial penetrance of *VPS13B* homozygous mutation has been hypothesized to be a reason for the variability in autism association and cognitive involvements [21]. Herein, we suggest an alternative hypothesis that points to the extent of injury and fragmentation of Golgi structures, in the tissues including the brain, as an important contributor to the differential functional impact and variability in neurodevelopmental findings in CS patients. This assumption is supported by the knowledge that the Golgi peripheral membrane proteins contribute to Golgi integrity and proper orientation and consequently its normal subcellular function in protein and lipid post-translational modification. In addition, the glycosylation defects in CS have been presumed to be due to Golgi disorganization secondary to alteration in the transmembrane VPS13B protein [22]. The hypothetical role of VPS13B in Golgi-mediated vesicle trafficking and endolysosomal transport has been recently verified in VPS13BΔEx3/ΔEx3 mice model [23]. This model demonstrated the occurrence of Golgi apparatus mislocalization and its aberrantly configured membrane stacks. The authors provided experimental evidence for the role of VPS13B in the transport of Golgi-mediated vesicles during the formation of spermatid’s acrosome.

The exciting molecular result, in this report, is the sequencing finding of the *NAPB* splice-site pathogenic variant, which recessively segregates with the phenotype in family members. *NAPB*-related neurodevelopmental disorder (*NAPB*-related NDD) is characterized mainly by EOEE and seems of ultra-rare occurrence; to date, it was documented in only four independent families and in association with loss of function mutations, two nonsense mutations [11, 12], and a recent splice-site mutation [13]. Our report is the 5th family worldwide.

There is a clinical overlap between *NAPB*- and *VPS13B*-associated neurodevelopmental features of intellectual disability, developmental delay, and autistic spectrum; however, the EOEE characterizes the *NAPB* phenotype.

Epilepsy has been described as an uncommon feature and is, generally, poorly characterized within CS patients. In the present report, epilepsy was significantly prominent and of variable severity among the triplets. The three probands started the seizures at the age of 6 months; however, proband A has the most intractable and predominantly nocturnal epilepsy phenotype with the highest frequency of seizures. The epileptic events in proband B were equally distributed throughout wakefulness and sleep and of moderate severity. Proband C has the mildest course of epilepsy. A variable response to antiepileptic drugs defines an additional seizure-related variability. The seizures previously described in a few independent CS patients were characterized by an older age of onset between 18 and 24 months, benign courses, and being smoothly manageable by antiepileptic medications. The reported EEGs recorded a spike-wave epileptic discharge that tends to be unilateral, prevails over the temporal-parietal-occipital regions, and is continuous during slow waves of sleep (CSWS) [24]. In our patients, the EEG demonstrated multifocal discharges supporting a previously unrecognized CS-brain phenotype of multifocal neonatal-onset epilepsy. The causal role of *NAPB*’s mutations is supported by the documented association of altered SNAP-beta protein with early-onset multifocal epileptic encephalopathy (EOEE) in patients with unexplained neurodevelopmental disorders [11–13].

The present pathogenic *NAPB* variant expands the phenotypic spectrum of Cohen syndrome and likely explains a role in the associated prominent neurodevelopmental behavioral phenotype*.* The SNAP-beta has been recognized in the pathways (http://PathCards.genecards.org) involved in intra-Golgi traffic, Golgi-derived vesicle trafficking and transport, and Golgi-to-ER retrograde transport. In the same direction, VPS13B co-localizes with Golgi matrix proteins and demonstrated a role in Golgi integrity [9]. This pays the attention to the common role of VPS13B and SNAP-beta proteins in Golgi-medicated vesicular transport.

## Conclusion

The family described here presents the first co-occurrence of CS and NAPB-related neurodevelopmental disorder in triplets from a consanguineous family. This report underscores the importance of studying the role of the Golgi membrane and functionally related proteins in the development of neurodevelopmental and behavioral disturbance disorders and adds to the ever-expanding landscape of genetic causes of early-onset syndromic non-channelopathy epilepsy.

Our family acknowledges the uniform finding of absent neutropenia in Arab CS patients, reported so far. Sexual arousal and aggressiveness that were significantly prominent and notably variable between the triplets expanded the clinical spectrum of the diseases.

## Data Availability

The exome sequencing data of this family is not publicly available. Inquiries to access the data can be made to the corresponding author.

## Footnotes

1 https://varsome.com

2 http://grch37.ensembl.org/Tools/VEP

3 http://www.homozygositymapper.org/HM/93179
